# The Maintenance and Collapse of Sense of Order: A Longitudinal Study of System Justification Mechanisms Among Chinese Youth

**DOI:** 10.1002/brb3.71604

**Published:** 2026-07-23

**Authors:** Hanqiang Li, Liwen Chen

**Affiliations:** ^1^ Faculty of Social Sciences and Liberal Arts UCSI University Kuala Lumpur Malaysia; ^2^ College of Tourism and Historical Culture Chizhou University Chizhou City China

**Keywords:** depression, meritocratic beliefs, system justification theory, trust, youth

## Abstract

**Background:**

Against the background of increasing competition and expanding inequality in modern society, individuals are motivated to maintain the legitimacy and stability of existing social systems. Within this process, meritocratic beliefs, trust, and depression together constitute a dynamic mechanism through which individuals maintain psychological stability during system justification processes. However, most existing studies examining the relationships among these three variables have relied on cross‐sectional designs or traditional cross‐lagged analyses, making it difficult to accurately capture their longitudinal psychological dynamics.

**Objective:**

The present study aimed to systematically examine the longitudinal predictive relationships among meritocratic beliefs, trust, and depression among Chinese young adults using multiwave longitudinal data.

**Methods:**

The data used in the present study were derived from the three‐wave longitudinal surveys conducted by the China Family Panel Studies in 2018, 2020, and 2022. After screening for missing values and age restrictions, the final sample included 1489 valid participants between the ages of 14 and 35. Data analysis was conducted using R Studio and the RI‐CLPM.

**Conclusion:**

At the level of stable between‐person traits, higher trust and stronger meritocratic beliefs were both significantly associated with lower long‐term baseline levels of depression. Trust and meritocratic beliefs also demonstrated a significant positive association. At the level of within‐person dynamic fluctuations, trust at the previous wave significantly and positively predicted subsequent meritocratic beliefs, whereas depression at the previous wave significantly and negatively predicted subsequent meritocratic beliefs. Meritocratic beliefs did not significantly predict later changes in either trust or depression.

## Graphical abstract

This three‐wave RI‐CLPM study found that higher trust and stronger meritocratic beliefs were associated with lower depression. Trust positively predicted later meritocratic beliefs, whereas depression negatively predicted them over time.

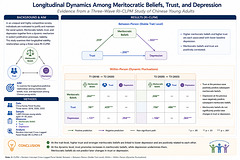

## 1 Introduction

Against the background of increasing competition and expanding inequality in modern society, individuals must confront not only material disparities in resources but also the psychological task of continuously explaining why society operates in its current form and why they occupy their present social position (Jost et al. 2018). System Justification Theory proposes that individuals possess a fundamental psychological motivation to maintain the legitimacy and stability of existing social systems. People tend to believe that society remains generally orderly, predictable, and to some extent fair (Osborne et al. 2019). This rationalization of the social system can reduce existential anxiety arising from uncertainty and help individuals preserve hope and a sense of meaning regarding future action. Within this process, meritocratic beliefs and social trust together constitute an important psychological foundation for maintaining a sense of social order (Geng et al. 2025). When individuals believe that effort can lead to reward, they are also more likely to believe that public institutions and the behavior of others are reliable. In general, if individuals perceive social rules as fair, they are more likely to regard the operation of society as trustworthy. This broader perception of order and reliability may significantly buffer the psychological threat generated by structural social pressures (Låftman et al. 2024).

Accordingly, the deterioration of mental health may reflect not only impaired emotional functioning but also the weakening of an individual's original sense of social order (Teymoori et al. 2017). When individuals repeatedly experience imbalance between effort and reward or encounter blocked opportunities for social mobility, these experiences may gradually undermine their belief in social fairness. Individuals may begin to question whether existing rules are genuinely effective, which may subsequently affect both physical and psychological health (Siegrist 1996). Under such conditions, the rationalization of the social system becomes increasingly difficult to maintain. Trust in institutions and other people may decline accordingly, and the social environment may come to be perceived as unpredictable, manipulative, or even hostile (Oishi et al. 2011). At the same time, because system justification mechanisms serve important psychological functions related to anxiety reduction and the maintenance of perceived control, the collapse of these mechanisms may deprive individuals of stable expectations regarding the future. Individuals may also become more likely to attribute failure either to personal inadequacy or to a fundamentally disordered external world. This process may generate persistent helplessness, self‐denial, and psychological exhaustion (Jost and Hunyady 2003), which may ultimately increase the risk of depression. From this perspective, meritocratic beliefs, social trust, and depression are jointly embedded within a broader process through which individuals cognitively rationalize and psychologically adapt to the social system. Together, these constructs reflect a dynamic mechanism through which individuals maintain psychological stability and a sense of meaning within unequal social structures.

However, although meritocratic beliefs, trust, and depression are theoretically characterized by closely interconnected dynamic relationships, existing empirical studies have largely relied on cross‐sectional designs or traditional cross‐lagged analyses. As a result, the strict longitudinal relationships underlying this developmental process remain insufficiently understood. The present study therefore introduces the Random Intercept Cross‐Lagged Panel Model (RI‐CLPM) to examine the longitudinal predictive relationships among these three core variables using multiwave longitudinal data. By extracting and controlling for stable random intercepts that exist between individuals, the present study is able to focus more directly on pure within‐person fluctuations. This shift in analytical perspective may help clarify the temporal relationships associated with the breakdown of system justification mechanisms at the empirical level. It may also provide a scientific basis for developing more precise psychological intervention strategies within highly competitive social environments.

## 2 Literature Review

### 2.1 Trust and Meritocratic Beliefs

Based on System Justification Theory, individuals tend to justify existing social arrangements in order to perceive themselves as living within a “fair” system (Jost et al. 2019). Meritocratic beliefs represent a system‐justifying ideology that increases the perceived legitimacy of social stratification. Trust is likewise regarded as an expression of institutional legitimacy. Using a trust game paradigm, Samson (2018) found that under conditions of income inequality, high‐income individuals exhibited greater trust toward other high‐income individuals, whereas low‐income individuals showed higher trust toward high‐income individuals. This pattern of outgroup trust was consistent with system justification motives. Geng et al. (2025) analyzed data from 84,638 participants across 57 societies using the World Values Survey. They found that societies with stronger endorsement of meritocratic beliefs exhibited higher levels of institutional trust at the societal level. At the individual level, individuals with stronger meritocratic beliefs showed greater trust in public institutions only in societies with high levels of economic freedom. The opposite pattern emerged in countries with low economic freedom. Greenwood‐Hau (2021) analyzed data from the United States and found that the belief that “hard work explains income inequality,” which represents one component of meritocratic ideology, was positively associated with trust in government. Most of the studies discussed above were based on cross‐sectional associations. Only limited experimental evidence, such as Samson's trust game experiment, examined trust as a dependent variable. In economically free and democratic societies, meritocratic beliefs are generally associated with higher institutional trust. However, this association may weaken or even reverse in countries characterized by highly developed welfare systems and dominant egalitarian values (Geng et al. 2025). In collectivist cultures, individuals may rely more heavily on interpersonal trust than institutional trust. As a result, empirical findings may not always be consistent (Xin and Pearce 1996).

From the opposite perspective, distrust toward governments and authoritative institutions, often reflected in conspiracy beliefs, has also been shown to be significantly negatively associated with elitist beliefs (Zhao et al. 2025). This conclusion has been further supported by multiple empirical studies and theoretical frameworks (Nera et al. 2026; Kishishita 2025). From the perspective of cognitive structure and system justification, the core premise of elitism lies in affirming the transparency and fairness of the macro‐level system of resource allocation. Individuals who endorse elitist beliefs tend to believe that personal talent and effort can be translated into corresponding socioeconomic rewards according to established social rules (Son Hing et al. 2011). In contrast, individuals who are inclined toward conspiracy beliefs typically assume that society is secretly controlled by a small group of selfish and manipulative elites. They regard public institutions and selection procedures as superficial mechanisms that conceal substantive resource monopolization and rent‐seeking behaviors (Imhoff and Bruder 2014). Consequently, when individuals increasingly perceive public institutions as unreliable or even malicious, they are more likely to attribute observed structural inequality to systemic corruption rather than to differences in personal ability or effort.

### 2.2 Depression and Meritocratic Beliefs

From the perspective of System Justification Theory, meritocratic beliefs may reduce anxiety arising from perceived inequality and thereby provide psychological reassurance for disadvantaged groups (Hadarics et al. 2021). Endorsing such beliefs implies that individuals perceive themselves as having control over their future outcomes, which may enhance self‐esteem and psychological resilience. Several studies have shown that meritocratic beliefs at both the individual and cultural levels are positively associated with mental health. Hadarics et al. (2021) analyzed survey data from the European Union and found that meritocratic beliefs were positively associated with subjective well‐being, including positive affect and self‐esteem. This relationship was particularly pronounced among low‐income groups and in countries with higher levels of income inequality. McCoy et al. (2013) reported that among low‐status women in the United States, endorsement of meritocratic beliefs was positively associated with self‐esteem and physical health. This relationship was mediated by perceived control and functioned as a psychological buffer. Although these studies were cross‐sectional and did not directly assess depressive symptoms, their findings suggest that meritocratic beliefs may be negatively associated with psychological distress. Silva et al. (2024) reviewed multiple studies on belief in a just world, which is conceptually related to meritocratic ideology. They consistently found that stronger fairness‐justifying beliefs were associated with lower levels of depression and anxiety. Their review also included more direct evidence. For example, among Chinese adolescents, belief in a just world was significantly associated with lower levels of depression (Feng et al. 2024).

However, meritocratic beliefs do not always produce positive psychological outcomes. When individuals fail to obtain the rewards they believe they deserve within a meritocratic culture, internalized meritocratic ideology may generate frustration and subsequently increase psychological distress and depression (Biström and Mollwing 2026). Recent studies have suggested that meritocratic ideology within educational systems may intensify self‐blame among students who experience failure and may contribute to persistent psychological suffering. These findings indicate that meritocratic culture can be psychologically harmful under certain conditions (Biström and Mollwing 2026). Current research on the negative psychological consequences of meritocratic beliefs generally emphasizes their conditional and context‐dependent nature. Meritocratic beliefs do not inherently produce psychological distress. Their negative effects tend to emerge under specific structural conditions. This pattern is especially evident when individuals repeatedly encounter failure despite sustained effort, when opportunities for social mobility are restricted, or when there is a persistent discrepancy between expected and actual rewards (Biström and Mollwing 2026; Efird et al. 2023; Sandel 2020). Under such circumstances, individuals may attribute structural inequality to personal inadequacy, which may further intensify depression and psychological exhaustion. Consequently, many studies primarily highlight the risks associated with frustrated meritocracy rather than suggesting that meritocratic beliefs are inherently detrimental to mental health.

Overall, existing evidence more strongly supports the view that meritocratic beliefs exert predominantly positive effects on mental health. Their protective function may reverse only under particular social conditions and prolonged experiences of failure. In broader social contexts, the sense of order provided by meritocratic beliefs generally continues to serve a relatively stable psychological protective function. Even when reality is not entirely fair, believing that “effort can lead to reward” may still reduce existential anxiety and help individuals maintain hope and motivation for future action (Jost and Hunyady 2003).

### 2.3 Trust and Depression

Research over the past decade has consistently shown a statistical association between levels of trust and depressive symptoms, although the direction of this relationship is sometimes complex. In evaluating the strength of causal evidence, most existing studies are observational. Longitudinal designs with more than three waves and natural experiments are generally considered more reliable sources of evidence. Although randomized controlled trials targeting trust are relatively limited, several public health intervention studies have shown that strengthening community connectedness and mutual support can improve psychological well‐being. These findings indirectly suggest that increasing trust may help prevent depression. For example, Liu et al. (2025) found among Chinese adolescents that individuals with lower baseline trust had a higher future risk of depression after controlling for multiple family risk factors. Låftman et al. (2024) conducted a prospective study among Swedish adolescents and found that adolescents with higher levels of generalized trust at ages 15–18 reported significantly lower levels of depression and anxiety symptoms at ages 20–21. Institutional trust showed no significant effect after controlling for generalized trust and related factors. Gylfason (2025) analyzed longitudinal data from the Icelandic national health surveys conducted in 2007 and 2009. The study found that both interpersonal trust and generalized trust were associated with reduced depressive symptoms after controlling for prior depression and physical health. The predictive effect of interpersonal trust was stronger. However, several findings suggest that the effect of trust depends on social context. Adjaye‐Gbewonyo et al. (2018) analyzed three‐wave panel data from the National Income Dynamics Study in South Africa. They found an interaction between individual trust and community trust. When the overall level of community trust was extremely low, higher individual trust was associated with more depressive symptoms. The authors argued that in highly distrustful environments, excessive trust may expose individuals to repeated interpersonal betrayal and disappointment, thereby increasing depression.

Taken together, the available evidence provides relatively strong support for the causal pathway from trust to depression. Multiple longitudinal studies have shown that individuals with lower baseline trust are more likely to develop depression in the future after controlling for initial depressive symptoms (Låftman et al. 2024). Some mediating evidence has also been identified through mechanisms involving social support and emotion regulation. In contrast, the pathway from depression to trust has received only indirect support. For example, cross‐lagged studies and short‐term diary studies have shown that depressive states may reduce perceived social support (Han et al. 2025). This pathway is consistent with theories of depressive withdrawal and subjective cognitive bias. Individuals with depression may misinterpret the intentions of others and experience greater difficulty establishing trusting relationships. Overall, the evidence for both directions can be considered moderately strong, although definitive causal proof remains limited.

### 2.4 Summary

Based on System Justification Theory, meritocratic beliefs function as a central system‐justifying ideology that encourages individuals to rationalize existing systems of social stratification and resource allocation. Individuals who endorse such beliefs tend to perceive the rules governing society as fundamentally fair. This internal affirmation and maintenance of macro‐level social order may directly enhance trust in public institutions and other people at the level of social cognition. At the emotional level, it may also alleviate existential anxiety arising from social inequality by providing individuals with a sense of control and order regarding the future. In turn, this process may reduce the risk of depression. Conversely, when system justification mechanisms fail under specific structural conditions, such as repeated frustration that undermines meritocratic beliefs or prolonged exposure to highly distrustful social environments, the erosion of social trust is often accompanied by the collapse of system‐legitimizing beliefs. Under these circumstances, individuals may lose their original psychological protection and become increasingly vulnerable to institutional distrust or self‐blame, which may ultimately intensify psychological exhaustion. Within the framework of system justification theory, meritocratic beliefs, therefore, function both as a cognitive foundation that sustains trust and as a psychological defense mechanism against depression. Together, trust, meritocratic beliefs, and depression constitute an interactive framework that helps explain how individuals psychologically adapt to unequal social structures.

## 3 Research Method

### 3.1 Data Source

The data used in this study were obtained from the China Family Panel Studies (CFPS), a nationally representative, large‐scale, and multidisciplinary longitudinal survey conducted by the Institute of Social Science Survey at Peking University. CFPS is widely recognized for its high academic reputation and methodological rigor both within China and internationally (Jin et al. 2024; Lai et al. 2022). Initiated in 2010 with a baseline survey, CFPS follows up with participants every 2 years. The project systematically documents and reflects the profound social, economic, demographic, educational, and health‐related transformations taking place in contemporary China through long‐term tracking of sample households across 25 provinces, municipalities, and autonomous regions. Funded by Peking University, the CFPS design draws extensively on best practices from leading international social survey projects, aiming to provide comprehensive and high‐quality data for academic research and public policy analysis. The present study utilizes data from the 2018, 2020, and 2022 survey waves. The use of these data have been approved by the CFPS research team, and therefore no additional institutional ethical review was required.

### 3.2 Measurement Selection

The present study involved three variables, including meritocratic beliefs, trust, and depression. In the formal analysis, data from 2018 were used as the baseline for conducting confirmatory factor analysis. Items with factor loadings below 0.40 were removed to ensure the minimum level of reliability of the measurement model.

To measure individuals’ meritocratic beliefs, items were selected from the social values section of the CFPS questionnaire. Specifically, four items were used: “Wealth reflects personal achievement,” “Hard work pays off,” “Intelligence and talent are rewarded,” and “There are many opportunities to improve one's standard of living.” Each item was rated on a five‐point Likert scale. These items capture the internalization of meritocratic beliefs across three dimensions—the process of striving, the outcomes of distribution, and the broader social environment. Thus, combining these four items into a composite indicator provides a valid and contextually appropriate measure of meritocratic beliefs in Chinese society. The overlap between these items and those used in prior studies further supports the validity and suitability of this selection (Cho et al. 2023; García‐Sierra 2023). The Cronbach's α coefficients for this scale in 2018, 2020, and 2022 were 0.93, 0.92, and 0.99, respectively.

Trust was measured using the trust scale from the CFPS dataset. This scale consists of six items rated on a 10‐point scale, assessing three dimensions of trust: Particularized Trust (parents and neighbors), Generalized Trust (strangers and Americans), and Institutional Trust (local government officials and doctors). The items include trust in parents, neighbors, Americans, strangers, local government officials, and doctors. Based on factor loadings, two items, trust in parents and trust in Americans, were removed from the analysis. The Cronbach's α coefficients for this scale were 0.75, 0.78, and 0.83 in 2018, 2020, and 2022, respectively.

Depression was assessed using the abbreviated version of the Center for Epidemiologic Studies Depression Scale (CES‐D) included in the CFPS. The CES‐D is one of the most widely used instruments internationally for screening depressive symptoms. It is not intended for clinical diagnosis but rather for the rapid evaluation and identification of the frequency and severity of recent depressive symptoms in the general population. The original CES‐D contains 20 items rated on a four‐point scale. The CFPS adapted this scale into an eight‐item short form to efficiently screen participants’ depressive symptoms. The retained items include feeling depressed, feeling that everything is an effort, poor sleep, feeling happy, feeling lonely, living a happy life, feeling sad, and feeling that life is not worth living. Two reverse‐coded items were removed based on factor loadings. The Cronbach's α coefficients for the 2018, 2020, and 2022 datasets were 0.95, 0.96, and 0.97, respectively.

### 3.3 Sample Selection and Missing Data Treatment

The sample in the present study was drawn from individuals aged 14–35 years in the CFPS database. Due to the long time span of the study and substantial sample attrition, the initial dataset contained a relatively large amount of missing data. Participants with > 5% missing data were excluded from the analysis. After the screening procedure, a total of 1489 valid youth participants were retained for model construction. The remaining missing data were handled using full information maximum likelihood (FIML). FIML estimates model parameters by using all available observed data from each individual case to compute the likelihood function for that participant. For cases with missing values, the likelihood function is calculated based only on the available observed data. Global model parameters are then estimated by maximizing the combined likelihood functions across all participants.

### 3.4 Research Tools

This study employed R Studio for model construction, data processing, and statistical analysis, while SPSS was used for initial data cleaning and preprocessing, including the removal of participants who did not meet the age criteria or had excessive missing data. Given the increasing criticism and methodological limitations associated with the traditional Cross‐Lagged Panel Model (CLPM) (Littlefield et al. 2022; Grimm et al. 2021), the present study adopted the RI‐CLPM for model estimation. Compared with the CLPM, the RI‐CLPM more effectively separates and controls for stable between‐person differences, allowing for a more precise examination of within‐person developmental changes and reciprocal associations among variables over time (Burns et al. 2020). By introducing random intercepts, the RI‐CLPM captures time‐invariant individual differences, thereby ensuring that the cross‐lagged paths primarily reflect intraindividual fluctuations across measurement waves. This approach aligns more closely with developmental psychology's emphasis on within‐person processes and longitudinal change (Hamaker et al. 2015).

Additionally, the study employed the Maximum Likelihood Estimation with Robust Standard Errors (MLR) method for parameter estimation. MLR is a widely used estimation technique in structural equation modeling (SEM) designed to handle violations of the multivariate normality assumption by scaling and adjusting the chi‐square test statistic, thus providing more accurate assessments of overall model fit when data exhibit non‐normality.

## 4 Research Results

### 4.1 Demographic Characteristics of the Sample

Table 1 presents the demographic characteristics of the participants, including age, gender, and the mean scores of the study variables across survey years. The results indicate that the three focal variables remained relatively stable over time, with no substantial fluctuations observed across measurement waves.

**TABLE 1:** Demographic information statistics table.

	*N* = 1489		
**Gender**	***n* (%)**		
Male	710 (47.68%)		
Female	779 (52.32%)		
**Age (2018)**			
14–18	264 (17.73%)		
19–22	197 (13.23%)		
23–26	236 (15.85%)		
27–30	355 (23.84%)		
31–35	437 (29.35%)		
	**Wave 2018**	**Wave 2020**	**Wave 2022**
	Mean ± SD		
**Trust**	5.213 ± 1.580	5.569 ± 1.445	5.467 ± 1.500
**Depression**	1.503 ± 0.436	1.546 ± 0.480	1.574 ± 0.502
**Meritocratic beliefs**	3.786 ± 0.536	3.783 ± 0.509	3.832 ± 0.549

### 4.2 Correlation Analysis Results

**FIGURE 1:**
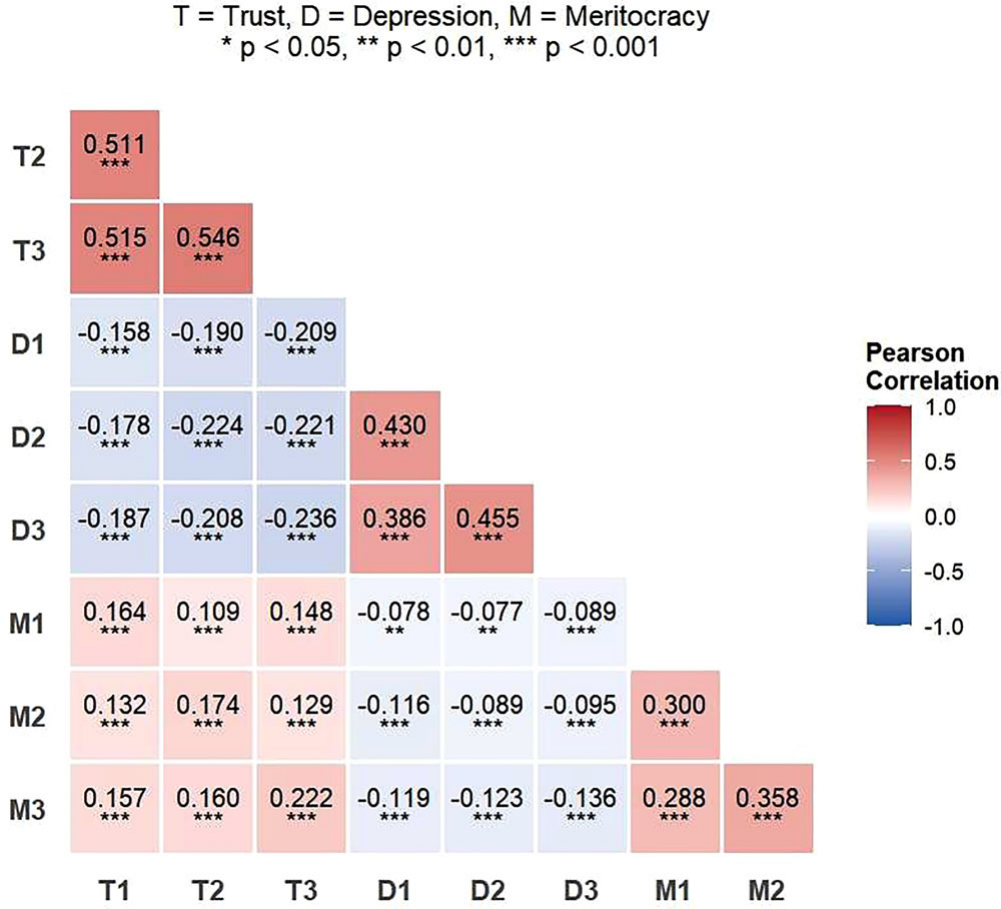
Correlation matrix of variables.

The Pearson correlation matrix (Figure 1) revealed the interrelations among trust (T), depression (D), and meritocratic beliefs (M) across the three time points.

### 4.3 Measurement Invariance Testing

To ensure the reliability of the longitudinal cross‐lagged analysis, the present study first conducted a systematic assessment of the measurement invariance of the three latent variables, including trust, depression, and meritocratic beliefs, across different time points (T1, T2, and T3) before testing the core structural pathways. Configural invariance models, metric invariance models that constrained factor loadings to be equal across waves, and scalar invariance models that further constrained item intercepts to be equal across waves were sequentially constructed and compared. Measurement invariance was primarily evaluated based on changes in fit indices, including ΔCFI and ΔRMSEA.

The results indicated that, relative to the configural invariance model (CFI = 0.926, RMSEA = 0.032), the metric invariance model showed only a slight decrease in CFI (ΔCFI = −0.002), whereas RMSEA demonstrated no substantial increase (ΔRMSEA < 0.001). These findings suggest that the observed indicators of the core latent constructs maintained relatively stable explanatory strength across the three waves of data. In the subsequent scalar invariance test, the scalar invariance model showed a ΔRMSEA of 0.003 and a ΔCFI of −0.019 relative to the metric invariance model, both of which remained within the acceptable threshold of 0.02. Taken together, the measurement invariance results provided a reliable methodological foundation for the subsequent longitudinal analyses. Detailed results of the measurement invariance tests are presented in Table 2.

**TABLE 2:** Fit indices for longitudinal measurement invariance models.

Model	CFI	TLI	RMSEA	SRMR	ΔCFI	ΔRMSEA
Configural invariance	0.926	0.915	0.032	0.044	—	—
Metric invariance	0.924	0.915	0.032	0.045	−0.002	< 0.001
Scalar invariance	0.905	0.897	0.035	0.048	−0.019	0.003

### 4.4 Analysis of the Sample Attrition Mechanism

Because the present study employed a three‐wave longitudinal design, a certain degree of sample attrition was unavoidable. At baseline in 2018, the number of participants who met the age requirements was 11,711. After excluding invalid cases with excessive missing data, the final valid sample at T3 consisted of 1489 participants, yielding a retention rate of 12.7%. Given the substantial number of excluded cases, an attrition analysis was conducted to examine whether the missing data mechanism violated the missing at random (MAR) assumption. To reduce the influence of multicollinearity and accidental significance, the present study first calculated the baseline mean scores of trust, depression, and meritocratic beliefs at T1. Independent‐samples *t* tests indicated that there was no significant difference in baseline overall depression levels between retained participants and attrited participants (*t* = −0.69, *p =* 0.487).

In addition, demographic variables, including age and gender, together with the baseline mean scores of the three core constructs, were simultaneously entered into a binary logistic regression model to predict the probability of attrition at the third wave. The regression results showed that none of the core variables or age significantly predicted sample attrition (*p* > 0.05). Only baseline gender significantly predicted attrition probability (*p* = 0.044). Detailed results are presented in Table 3. These findings suggest that sample attrition in the present study was primarily associated with demographic characteristics rather than systematic differences in the initial levels of the core observed variables. Therefore, the missing data mechanism was considered consistent with the MAR assumption. Accordingly, gender was included as a control variable in the subsequent model construction. FIML was applied to estimate model parameters and to provide approximately unbiased parameter estimates under the MAR assumption.

**TABLE 3:** Logistic regression predicting sample attrition using baseline characteristics.

Baseline variables (T1)	*β*	S.E.	*z*	*p*
Intercept	−0.024	0.198	−0.123	0.903
Age	−0.003	0.003	−0.900	0.368
Gender	−0.020	0.010	−2.015	0.044
Trust	−0.022	0.013	−1.600	0.110
Depression	0.028	0.046	0.604	0.546
Meritocratic beliefs	−0.021	0.039	−0.540	0.589

### 4.5 Random Intercept Cross‐Lagged Panel Model

The present study used the R package lavaan to construct an RI‐CLPM. The model demonstrated satisfactory fit indices, including CFI = 0.918, TLI = 0.910, RMSEA = 0.031 (0.029, 0.032), and SRMR = 0.045. All indices met the criteria for good model fit, indicating a high degree of consistency between the theoretical model and the observed data and providing adequate support for the research hypotheses. At the level representing stable individual traits (see Table 4), the random intercepts of trust and meritocratic beliefs showed a significant positive correlation (*r* = 0.259, *p* = 0.025). This finding suggests that individuals who were dispositionally more likely to trust others also tended to exhibit higher levels of meritocratic beliefs at the trait level. The correlation between the random intercepts of depression and trust was significantly negative (*r* = −0.503, *p* < 0.001). In addition, the correlation between the random intercepts of depression and meritocratic beliefs was also significantly negative (*r* = −0.200, *p* < 0.01).

**TABLE 4:** Correlation data of random intercept.

	*r*	S.E.	*z*	*p*
Trust (RI)—depression (RI)	−0.503	0.013	−5.950	***
Trust (RI)—meritocratic beliefs (RI)	0.259	0.020	2.247	*
Meritocratic beliefs (RI)—depression (RI)	−0.200	0.004	−2.593	**

*Note*: *For p <.05, ** for p <.01, *** for p <.001, the same applies below.

**FIGURE 2:**
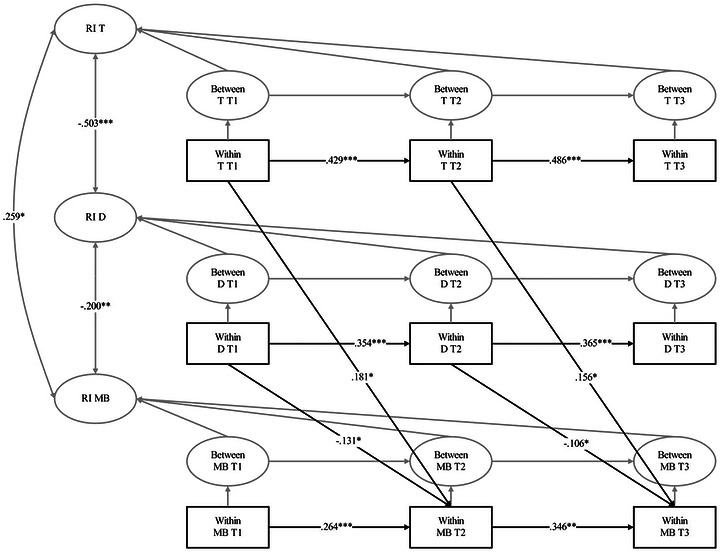
Cross‐lagged path diagram.

According to the regression results of the RI‐CLPM (see Figure 2), all three latent variables demonstrated significant and moderate‐to‐strong autoregressive effects within the within‐person dynamic processes. All autoregressive path coefficients were > 0.20 and statistically significant at *p* < 0.01. These findings indicate that an individual's state at one time point strongly predicted the same construct at the subsequent time point.

With regard to the cross‐lagged effects, the model results revealed that trust and depression jointly predicted subsequent meritocratic beliefs, whereas no reciprocal relationships were identified. Trust at the previous wave significantly and consistently positively predicted meritocratic beliefs at the following wave. The standardized estimate from T1 to T2 was 0.181 (*p* = 0.032), and the estimate from T2 to T3 was 0.156 (*p* = 0.024). In contrast, depression at the previous wave significantly negatively predicted subsequent meritocratic beliefs. The estimate from T1 to T2 was −0.131 (*p* = 0.033), whereas the estimate from T2 to T3 was −0.106 (*p* = 0.043). Aside from these two pathways predicting meritocratic beliefs, no additional significant cross‐lagged associations were observed in the model. Trust and depression did not demonstrate significant reciprocal within‐person cross‐temporal predictive effects. Similarly, meritocratic beliefs did not significantly predict subsequent levels of trust or depression. Detailed results for the autoregressive and cross‐lagged pathways are presented in Table 5.

**TABLE 5:** Autoregressive and cross‐lagged path parameter estimates.

Path direction	*β*	S.E.	*p*
T1 → T2			
Trust → Trust	0.429	0.062	***
Depression → Trust	−0.070	0.063	0.129
Meritocratic beliefs → Trust	0.062	0.229	0.225
Trust T1 → Depression	−0.071	0.036	0.143
Depression → Depression	0.354	0.059	***
Meritocratic beliefs → Depression	0.001	0.169	0.984
Trust → Meritocratic beliefs	0.181	0.017	0.032
Depression → Meritocratic beliefs	−0.131	0.019	0.033
Meritocratic beliefs → Meritocratic beliefs	0.264	0.068	***
T2 → T3			
Trust → Trust	0.486	0.093	***
Depression → Trust	−0.073	0.063	0.148
Meritocratic beliefs → Trust	0.074	0.373	0.378
Trust → Depression	−0.035	0.049	0.541
Depression → Depression	0.365	0.050	***
Meritocratic beliefs → Depression	−0.085	0.238	0.181
Trust → Meritocratic beliefs	0.156	0.018	0.024
Depression → Meritocratic beliefs	−0.106	0.017	0.043
Meritocratic beliefs → Meritocratic beliefs	0.346	0.129	0.002

**FIGURE 3:**
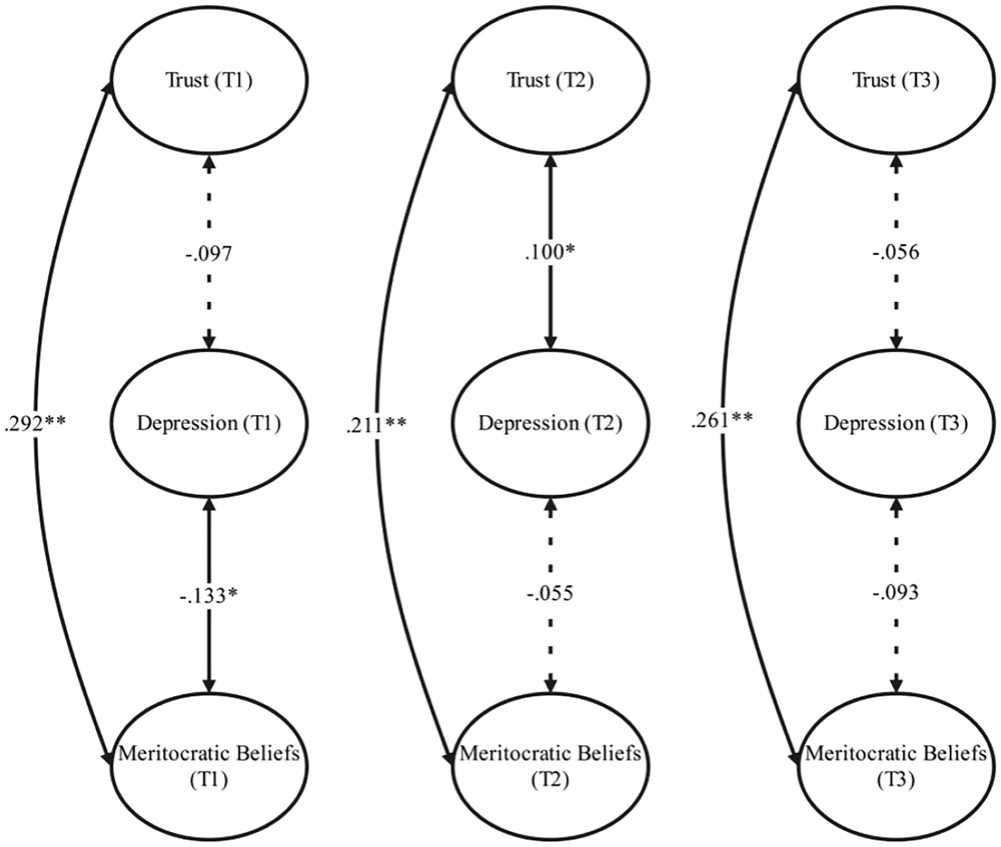
Latent within‐wave covariances.

After controlling for autoregressive effects, random intercepts, and cross‐lagged pathways, synchronous correlations among the variables were further examined. Trust and meritocratic beliefs demonstrated stable patterns of concurrent covariation. Significant positive synchronous correlations between these two variables were observed across T1, T2, and T3, with p values of 0.005, 0.004, and 0.002, respectively. The synchronous covariation patterns of the other variable pairs fluctuated across time. The negative synchronous association between depression and meritocratic beliefs was significant only at T1 (*p* = 0.016) and became non‐significant at later waves. In contrast, the negative synchronous association between trust and depression reached statistical significance only at T2 (*p* = 0.039). This association was marginally significant at T1 and completely non‐significant at T3. Detailed results for the synchronous correlations are presented in Table 6 and Figure 3.

**TABLE 6:** Synchronous correlations of latent variables within waves.

Wave	Pair of variables	*r*	S.E.	*p*
T1	Trust → Depression	−0.097	0.014	0.060
	Trust → Meritocratic beliefs	0.292	0.008	0.005
	Depression → Meritocratic beliefs	−0.133	0.003	0.016
T2	Trust → Depression	−0.100	0.011	0.039
	Trust → Meritocratic beliefs	0.211	0.005	0.004
	Depression → Meritocratic beliefs	−0.055	0.003	0.303
T3	Trust → Depression	−0.056	0.012	0.282
	Trust → Meritocratic beliefs	0.261	0.006	0.002
	Depression → Meritocratic beliefs	−0.093	0.003	0.062

### 4.6 Comparison with the Competing Model

**FIGURE 4:**
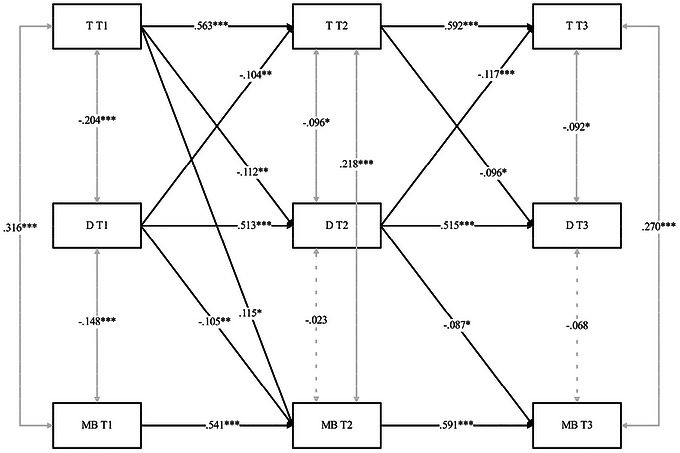
Path diagram of the traditional CLPM.

To further verify the necessity of separating stable between‐person traits from within‐person fluctuations over time, the present study constructed a traditional CLPM as a competing model (see Figure 4). Comparison of the fit indices clearly demonstrated that the target model incorporating random intercepts outperformed the competing model across all indicators. The traditional CLPM, which did not separate stable trait differences, showed relatively weaker model fit (CFI = 0.905, TLI = 0.896, RMSEA = 0.035, SRMR = 0.049). This finding suggests that the RI‐CLPM possesses stronger statistical advantages in balancing explanatory power and parsimony. To conduct a more rigorous statistical comparison, the present study performed a Satorra–Bentler scaled likelihood ratio test between the two nested models. From a mathematical perspective, the traditional CLPM is equivalent to constraining the variances and covariances of the random intercepts representing stable between‐person heterogeneity in the RI‐CLPM to zero. The test results confirmed that imposing these strong constraints significantly worsened model fit (*p* < 0.001). Therefore, adopting the RI‐CLPM rather than the traditional CLPM was justified for identifying the genuine longitudinal dynamic relationships among the core variables.

## 5 Discussion

### 5.1 Associations among Stable Individual Traits

At the random intercept level representing stable between‐person differences, the data revealed significant systematic associations among high levels of trust, strong meritocratic beliefs, and low baseline levels of depression. Individuals with higher trust baselines exhibited lower long‐term tendencies toward depression (*r* = −0.503, *p* < 0.001) and also demonstrated stronger meritocratic beliefs (*r* = 0.259, *p* < 0.05). In addition, stronger meritocratic beliefs were significantly associated with lower long‐term baseline levels of depression (*r* = −0.200, *p* < 0.01). The systematic associations identified at the random intercept level reflect a stable psychological structure that gradually develops through long‐term socialization processes. This structure may originate from the quality of early attachment relationships, persistent experiences of social support, and cognitive patterns accumulated through long‐term interactions with social institutions (Clifton et al. 2019). Individuals who more frequently experience others as reliable, social rules as consistent, and resource allocation as relatively fair during development are more likely to perceive the social world as fundamentally orderly (Janoff‐Bulman 1989). Individuals with higher levels of trust may be more likely to endorse meritocratic beliefs because both constructs share an underlying worldview assumption that the world is generally predictable, orderly, and governed by relatively stable rules (Wiederkehr et al. 2015). This cognitive orientation may encourage individuals to interpret stressful experiences in a more positive and controllable manner, thereby maintaining lower baseline levels of depression across broader time scales. In contrast, individuals who remain in chronically low‐trust environments may develop a stronger perception that social processes are fundamentally uncontrollable, making meritocratic beliefs more difficult to establish.

The findings suggest that trust, meritocratic beliefs, and depression together constitute a stable adaptive system characterized by a positive social orientation. However, the strength of the protective functions associated with these two psychological resources is not equivalent. The data indicated that trust showed a relatively strong negative association with baseline depression (*r* = −0.503). This effect suggests that trust may function as a more direct buffer against psychological exhaustion. Such protection may operate through the mediating role of high‐quality interpersonal relationships. Individuals with higher trust baselines are more likely to adopt tolerant and cooperative attitudes during long‐term social interactions, which may facilitate the development of healthier interpersonal networks (Malti et al. 2016). When confronted with life stress, these high‐quality interpersonal connections may function not only as substantial forms of social capital (Igarashi and Hirashima 2021) but also as important sources of stable psychological security. This process may help maintain depression risk at relatively low baseline levels across longer time scales (Gariépy et al. 2016). Although meritocratic beliefs also demonstrated a significant protective association with depression, the magnitude of this effect was comparatively moderate (*r* = −0.200). Meritocratic beliefs may help individuals maintain positive internal interpretations when facing adversity by providing a sense of order within a complex social environment, thereby reducing the likelihood of learned helplessness. The difference in the strength of these mechanisms suggests that, in maintaining psychological equilibrium, direct experiences of supportive social relationships may play a more fundamental role than perceptions of fairness within social rules.

### 5.2 Cross‐Lagged Path Analysis

In the RI‐CLPM, the random intercepts remove stable between‐person differences. The remaining paths therefore reflect only whether within‐person fluctuations relative to an individual's own long‐term average level persist over time. In many RI‐CLPM studies, autoregressive effects tend to weaken substantially or even become non‐significant, particularly for attitudes, emotions, and social cognitive variables, because short‐term changes in these constructs are often more strongly influenced by situational factors. However, in the present study, trust, depression, and meritocratic beliefs all demonstrated significant autoregressive effects of at least moderate magnitude. This finding indicates that the three variables not only exhibit stable between‐person differences but also possess substantial and relatively stable within‐person temporal inertia. In other words, when individuals become more trusting, more depressed, or more supportive of meritocratic beliefs than usual during a particular period, these deviations from their personal baseline levels continue to influence subsequent development. Existing cognitive and emotional states shape later patterns of information processing and behavioral responses. This continuity also suggests that the variables examined in the present study may gradually become internalized as relatively stable frameworks of social cognition.

Contrary to the original hypotheses, the only stable cross‐lagged effects identified in the model were unidirectional paths predicting meritocratic beliefs. This finding suggests that meritocratic beliefs, rather than depression, may function more as an outcome‐oriented cognition that dynamically adjusts according to individuals’ changing social experiences and psychological states. When individuals exhibited higher levels of trust than usual, they became more likely to endorse meritocratic beliefs at the subsequent stage (T1–T2: *β* = 0.181, *p* < 0.05; T2–T3: *β* = 0.156, *p* < 0.05). Theoretically, individuals are motivated to preserve the psychological perception that they live within a fair, controllable, and secure social world. As a result, they may consciously or unconsciously legitimize existing social institutions, even when such institutions are not always objectively beneficial to their own interests. Trust in other people and public institutions may allow individuals to feel that their efforts will not be unfairly exploited. Consequently, individuals may more naturally infer during subsequent cognitive evaluations that social resources are distributed fairly according to personal ability. Increases in trust may therefore reduce emotional doubt regarding the legitimacy of the social system, making individuals more likely to accept the underlying logic of meritocratic beliefs during the following stage. Under these conditions, individuals may not only more readily acknowledge the legitimacy of existing social stratification but may also continue to interpret potential systemic inequality primarily through standards of performance and achievement in order to maintain psychological security.

In contrast to the positive predictive effect of trust on meritocratic beliefs, the significant negative cross‐lagged path from depression to meritocratic beliefs indicates that when individuals experience higher levels of depression than their usual state during a given period, their subsequent endorsement of meritocratic beliefs tends to decline accordingly (T1–T2: *β* = −0.131, *p* < 0.05; T2–T3: *β* = −0.106, *p* < 0.05). According to Learned Helplessness and subsequent attributional models, individuals who repeatedly experience uncontrollable negative events tend to develop the expectation that behavior cannot effectively alter outcomes, gradually reducing their perceived sense of environmental controllability (Maier and Seligman 1976; Abramson et al. 1978). Depression is characterized not only by increased negative affect (Beck 2008) but also by low self‐efficacy, diminished perceived control, and pessimistic expectations regarding future outcomes (Abramson et al. 1989). These cognitive characteristics are fundamentally inconsistent with the core assumptions underlying meritocratic beliefs, because meritocratic beliefs emphasize a stable relationship between personal ability and social reward (Marvel 2025) and implicitly involve a strong sense of internal control. Consequently, when individuals experience elevated levels of depression, their subjective evaluations of social mobility opportunities and the effectiveness of personal effort may change, thereby reducing endorsement of meritocratic beliefs. For individuals experiencing depression, if feelings of powerlessness in reality cannot be interpreted as temporary and changeable conditions, continuing to believe that society operates entirely according to fair performance‐based allocation may further intensify negative self‐attribution and self‐denial. Under such circumstances, reduced endorsement of meritocratic beliefs, accompanied by greater emphasis on structural constraints, unequal opportunities, or external environmental factors, may function as a process of cognitive restructuring that alleviates the psychological burden associated with excessive internalization of failure.

Overall, meritocratic beliefs appear to function more as an outcome‐oriented form of social cognition at the within‐person dynamic level, shaped by other psychological states. Across both stages of the cross‐lagged analysis, trust and depression consistently predicted subsequent meritocratic beliefs, whereas meritocratic beliefs did not significantly predict later trust or depression. This finding suggests that individuals’ perceptions regarding the relationship between personal ability and social reward may change according to their current social experiences and psychological states. Traditional research has generally treated ideology and social values as important antecedents influencing individual psychological states (Geng et al. 2025; McCoy et al. 2013). However, the present findings suggest that psychological states themselves may continuously shape how individuals interpret the operating logic of social systems. This implies that judgments regarding whether society is fair, whether effort is effective, and whether social resources are distributed according to ability may partly derive from individuals’ current emotional experiences and social interaction patterns rather than solely from stable value systems.

### 5.3 Practical Implications

At the practical and clinical levels, the present study provides empirical evidence for optimizing mental health intervention models and improving the governance of broader social psychological climates. Based on the results of the cross‐lagged model, depression demonstrated a significant negative predictive effect on meritocratic beliefs (T1–T2: *β* = −0.131, *p* < 0.05; T2–T3: *β* = −0.106, *p* < 0.05), whereas the reverse predictive path from meritocratic beliefs to depression was not significant (T1–T2: *β* = 0.001, *p* = 0.98; T2–T3: *β* = −0.085, *p* = 0.18). This asymmetric relationship suggests that meritocratic beliefs cannot function as an effective psychological resource for alleviating depressive symptoms. Mental health practitioners should therefore avoid relying excessively on meritocratic narratives such as the assumption that effort necessarily leads to reward when conducting cognitive and behavioral interventions. From the perspective of psychopathology, one of the core cognitive impairments associated with depression involves reduced perceived control (Ulleberg et al. 2021), accompanied by a tendency to make internal and pessimistic attributions regarding negative events (Jiang 2024). Under such psychological conditions, emphasizing the fairness of the social system and the importance of individual agency may produce unintended iatrogenic effects. If macro‐level meritocratic ideology is directly incorporated into micro‐level psychological intervention, individuals experiencing depression may attribute their current difficulties to inherent personal deficiencies because they lack sufficient psychological efficacy to change their circumstances. This process of internalizing responsibility may fail to promote behavioral change and may instead intensify persistent psychological exhaustion.

In addition, the present study further distinguished stable between‐person associations from within‐person dynamic effects. Although trust and depression exhibited a significant negative association at the random intercept level, indicating that individuals with higher long‐term trust generally showed lower baseline levels of depression, the two variables did not demonstrate significant bidirectional predictive relationships at the within‐person cross‐lagged level. This finding suggests that the association between trust and depression may primarily originate from shared long‐term background factors, such as personality traits, developmental experiences, or enduring social environments, rather than from direct dynamic causal influences between short‐term fluctuations in psychological states. From the perspective of mental health intervention, these findings indicate that depression may be closely associated with the long‐term quality of social relationships, stable support systems, and sustained experiences within the social environment. Consequently, mental health promotion among young people should not focus exclusively on short‐term symptom management. Greater attention should also be directed toward the development of long‐term social support networks, improvement in interpersonal relationship quality, and cultivation of stable psychological security. Only when individuals remain within relatively trustworthy social environments over extended periods may deeper changes occur in both trust and psychological health.

## 6 Conclusion

The present study used large‐scale longitudinal tracking data to clarify the complex longitudinal relationships among trust, depression, and meritocratic beliefs among Chinese young adults through the RI‐CLPM. At the level of long‐term stable psychological structure, these three variables jointly constituted a stable adaptive system characterized by a positive social orientation. High baseline levels of trust and endorsement of meritocratic beliefs accumulated through long‐term socialization processes may function as important protective factors that help individuals resist psychological exhaustion and maintain lower levels of depression. However, within the dynamic process of intraindividual fluctuations over time, meritocratic beliefs did not function as antecedent variables shaping psychological health. Instead, they appeared to represent outcome‐oriented cognitions that continuously adjusted according to individuals’ changing social experiences and emotional states.

It should also be noted that meritocratic beliefs are not inherently beneficial buffering factors in the traditional sense. They not only failed to alleviate depressive symptoms within the intraindividual dynamic process, but under conditions of structural inequality they may even encourage individuals to internalize failure and legitimize the oppression they experience in reality, thereby producing further harm to psychological and physical health. Consequently, any psychological or social intervention developed on the basis of the present findings should be implemented with substantial caution. In both clinical intervention for youth depression and broader social governance, simplistic promotion of meritocratic discourse should be avoided. Greater emphasis should instead be placed on establishing stable long‐term social support networks and improving the sense of security within interpersonal relationships. Finally, it must be acknowledged that unavoidable sample attrition occurred across the three‐wave longitudinal tracking process. Because of limitations associated with the final effective sample size, the present study included only gender as a control variable during model construction. This limitation introduces the statistical risk of omitting other potential confounding variables, such as socioeconomic status and educational attainment. Future research should therefore examine this dynamic interactive mechanism using larger and more stable longitudinal samples while incorporating more comprehensive statistical controls and validation procedures.

## Author Contributions

**Hanqiang Li**: methodology, writing – original draft, writing – review and editing, validation, software, conceptualization, investigation, data curation. **Liwen Chen**: conceptualization, funding acquisition, writing – review and editing, methodology, supervision, resources.

## Funding

The article publication charge and related publication support were partially supported by the 2025 General Project of Anhui Provincial Higher Education Ideological and Political Work Innovation and Development Center (Grant No. AHSGSZX2025Y12), the 2026 University‐level Cultivation Project of the Higher Education Ideological and Political Work Quality Improvement Plan of Chizhou University (Grant No. SZ2026xjpy004), and the 2023 Teaching Quality Engineering Online Course Project of Chizhou University (Grant No. 2023XXSKC06).

## Ethics Statement

This study conducted a secondary analysis of de‐identified data from the CFPS. The CFPS project involves research on human participants and has been regularly reviewed and approved by the Peking University Biomedical Ethics Committee. The unified ethics approval number for the CFPS project is IRB00001052‐14010. Informed consent was obtained from all individual participants (or their parents/legal guardians in the case of minors) included in the survey by the CFPS project team prior to the data collection.

## Conflicts of Interest

The authors declare no conflicts of interest.

## Data Availability

The data that support the findings of this study are available from the China Family Panel Studies. The CFPS data are publicly available for research purposes upon registration and application through the official website of China Family Panel Studies (https://www.isss.pku.edu.cn/cfps/).
